# eRegistries: Electronic registries for maternal and child health

**DOI:** 10.1186/s12884-016-0801-7

**Published:** 2016-01-19

**Authors:** J. Frederik Frøen, Sonja L. Myhre, Michael J. Frost, Doris Chou, Garrett Mehl, Lale Say, Socheat Cheng, Ingvild Fjeldheim, Ingrid K. Friberg, Steve French, Jagrati V. Jani, Jane Kaye, John Lewis, Ane Lunde, Kjersti Mørkrid, Victoria Nankabirwa, Linda Nyanchoka, Hollie Stone, Mahima Venkateswaran, Aleena M. Wojcieszek, Marleen Temmerman, Vicki J. Flenady

**Affiliations:** Department of International Public Health, Norwegian Institute of Public Health, Pb 4404 Nydalen, N-0403 Oslo, Norway; Centre for Intervention Science in Maternal and Child Health (CISMAC), University of Bergen, Bergen, Norway; John Snow, Inc., Boston, MA USA; Department of Reproductive Health and Research, World Health Organization, Geneva, Switzerland; Faculty of Medicine, University of Oslo, Oslo, Norway; HeLEX - Centre for Health, Law and Emerging Technologies, Nuffield Department of Population Health, University of Oxford, Oxford, UK; Health Information System Programme (HISP) Vietnam, Ho Chí Minh, Vietnam; Department of Informatics, University of Oslo, Oslo, Norway; Department of Epidemiology and Biostatics, School of Public Health, College of Health Sciences, Makerere University, Kampala, Uganda; Mater Research Institute, The University of Queensland, Brisbane, Australia; International Stillbirth Alliance, Millburn, NJ USA

**Keywords:** Health systems, eRegistries, Women’s, Children’s and Adolescent’s Health, health surveillance, eHealth, mHealth

## Abstract

**Background:**

The Global Roadmap for Health Measurement and Accountability sees integrated systems for health information as key to obtaining seamless, sustainable, and secure information exchanges at all levels of health systems. The Global Strategy for Women’s, Children’s and Adolescent’s Health aims to achieve a continuum of quality of care with effective coverage of interventions. The WHO and World Bank recommend that countries focus on intervention coverage to monitor programs and progress for universal health coverage. Electronic health registries - eRegistries - represent integrated systems that secure a triple return on investments: First, effective single data collection for health workers to seamlessly follow individuals along the continuum of care and across disconnected cadres of care providers. Second, real-time public health surveillance and monitoring of intervention coverage, and third, feedback of information to individuals, care providers and the public for transparent accountability. This series on eRegistries presents frameworks and tools to facilitate the development and secure operation of eRegistries for maternal and child health.

**Methods:**

In this first paper of the eRegistries Series we have used WHO frameworks and taxonomy to map how eRegistries can support commonly used electronic and mobile applications to alleviate health systems constraints in maternal and child health. A web-based survey of public health officials in 64 low- and middle-income countries, and a systematic search of literature from 2005–2015, aimed to assess country capacities by the current status, quality and use of data in reproductive health registries.

**Results:**

eRegistries can offer support for the 12 most commonly used electronic and mobile applications for health. Countries are implementing health registries in various forms, the majority in transition from paper-based data collection to electronic systems, but very few have eRegistries that can act as an integrating backbone for health information. More mature country capacity reflected by published health registry based research is emerging in settings reaching regional or national scale, increasingly with electronic solutions. 66 scientific publications were identified based on 32 registry systems in 23 countries over a period of 10 years; this reflects a challenging experience and capacity gap for delivering sustainable high quality registries.

**Conclusions:**

Registries are being developed and used in many high burden countries, but their potential benefits are far from realized as few countries have fully transitioned from paper-based health information to integrated electronic backbone systems. Free tools and frameworks exist to facilitate progress in health information for women and children.

**Electronic supplementary material:**

The online version of this article (doi:10.1186/s12884-016-0801-7) contains supplementary material, which is available to authorized users.

## Background

Electronic health (eHealth) solutions, including mobile health technologies (mHealth), have the potential to improve quality of healthcare by addressing technical shortcomings embedded in health systems (Frame 1). Many eHealth initiatives in low and middle income countries (LMIC) have been fragmented [1, 2]. Now, global health agencies are moving towards more sustainable and holistic approaches for institutionalizing e- and mHealth into healthcare systems [1, 3–5].

**Frame 1:**
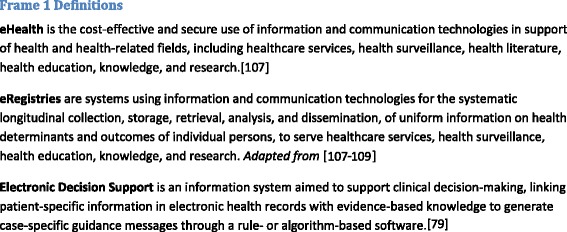
Definitions [79, 107–109]

The new World Bank/WHO/USAID Roadmap for Health Measurement and Accountability Post-2015 (MA4Health) underlined that *“Public health and clinical care cannot be delivered safely, with high quality, and in a cost-effective manner, without seamless, sustainable, and secure data and information exchanges at all levels of the health system”* [5]. An effective healthcare system must therefore entail a seamless common or interoperable digital thread for health information so that the full range of uses can be driven by, or contribute to, an *integrated backbone system* [4, 5]. In line with this, MA4Health has outlined the importance of having all health information system (HIS) development initiatives aligned with a single operational country platform for data and indicators by 2020.

With the new United Nation’s Global Strategy for Women’s, Children’s and Adolescent’s Health (UN Global Strategy) [6], the global community for mothers’ and children’s health is converging on post-2015 policies for integrated care for the health and survival of the mother and her baby alike. This brings together multiple recent initiatives such as *Strategies toward Ending Preventable Maternal Mortality* [7] and the *Every Newborn Action Plan* [8], representing an integrated continuum of community and facility health promotion and care from family planning, thru periconception, pregnancy, childbirth and postpartum, to the newborn and child. The potential of health systems focusing on the continuum of care cannot come to fruition without integrated eHealth solutions [9]. When any isolated silo keeps its information separate, potential synergies within the system are being squandered. eHealth is the most frequently mentioned emerging opportunity for maternal health among international researchers [10].

Better data on health status and quality of healthcare are crucial to address bottlenecks in achieving universal health coverage (UHC) and producing better policies for health. Traditional measures of points of contact, such as attending antenatal care or having a skilled birth attendant, are far from sufficient measures of having received quality care [11, 12]. Pertaining to this, the WHO and the World Bank accentuate that in monitoring UHC, the coverage of health interventions should be at the center of countries’ attention [13–15].

In the maternal and child health context, primary data on coverage of interventions is typically created when a woman is booked for antenatal care, and data on her health and the services she receives is subsequently added, retrieved and reported from her personal file over a continuum of community and facility services. But without eHealth in many settings, paper registers and patient folders make no timely and actionable data available for program management and policy development, and subsequent extraction of data from paper files results in poor quality data and underutilized health information [16–19]. Information on the individual woman meant to allow personalized care throughout pregnancy and childbirth is often neither easily accessed at follow up visits, nor shared between levels of care, or shared with women themselves to improve self-care. When information is shared, it is often not under robust governance to secure privacy and safety (Myhre et al: eRegistries: Governance for maternal and child health registries, submitted). Most public health data collection strategies are inefficient reporting chores, where care providers are viewed only as data collectors, and women only as data points. Not harvesting the data created and registered at the point-of-care, LMIC spend scarce resources on expensive data collection either by duplicate data entry from paper files, or undertaking household surveys to collect information from the population, with little and biased information of moderate validity on coverage of health interventions [20, 21, Flenady et al: eRegistries: Indicators for the WHO Essential Interventions for reproductive, maternal, newborn, and child health, submitted].

The UN Global Strategy has not formulated a specific eHealth component to support its activities [6], but such eHealth solutions will have to provide seamless and secure information following individuals across health system levels to serve the uniquely longitudinal focus on continuity and quality of care. It will also need to enable regional and national monitoring of coverage of health interventions delivered at the point-of-care. The «*integrated backbone systems*» [4, 5] that can deliver on both, are electronic health registries – eRegistries (Frame 1). Unlike health information system architectures that manage only aggregate data or clinical health records with unstructured text or forms, eRegistries are based on systematic and uniform data on pre-defined health outcomes and determinants, including care provision. This represents a database that can drive multiple e- and mHealth applications for health systems, individual care providers, and the individual clients and patients.

This eRegistries initiative, led by the Norwegian Institute of Public Health and the WHO Department of Reproductive Health and Research (RHR), with Queensland University, the University of Oxford, and the Health Information Systems Program Vietnam, aimed to develop a common framework of evidence, guidance and technical tools to facilitate the development and country implementation of eRegistries for reproductive, maternal, newborn and child health (RMNCH) in LMIC. eRegistries only have value if they can alleviate health system constraints hampering UHC. In this first paper of the eRegistries Series, we use the WHO frameworks and taxonomy for mHealth and health systems constraints in RMNCH to review what eRegistries, acting as the backbone HIS, can and should contribute to facilitate achievement of UHC of high quality care. We report on a systematic review of scientific literature from registries for RMNCH in LMIC, and on a survey of country readiness to develop eRegistries for RMNCH.

In the second paper (Flenady et al, submitted), we review the current availability of data and indicator gaps for monitoring and evaluation of coverage of the WHO Essential Interventions, Commodities and Guidelines for RMNCH [22]. We present the process and results in the harmonization and development of a suite of process (or coverage) and outcome indicators for use in eRegistries.

In the third paper (Myhre et al, submitted), we report the current status of ethical and legal issues pertaining to eRegistries in LMIC. Given the highly sensitive nature of RMNCH data, we assess existing privacy legislation, access, and data security practices and report on the development of a governance toolkit that outlines best practices for responsible data stewardship.

In the fourth and last paper (Frost et al: eRegistries: Architecture and free open source software for maternal and child health registries, submitted), we draw on the first three papers’ findings of identified needs, to report on the formulation of minimum criteria for free and open source software for eRegistries as the integrating backbone for HIS in RMNCH. We review potential systems and their functionalities, and report on the *eRegistries* application - a customizable point-of-care registry using WHO Essential Interventions care algorithms developed in DHIS2, the most commonly nationally deployed free and open source software health management information system [23].

## Methods

### Framework for eHealth in RMNCH

**Fig. 1:**
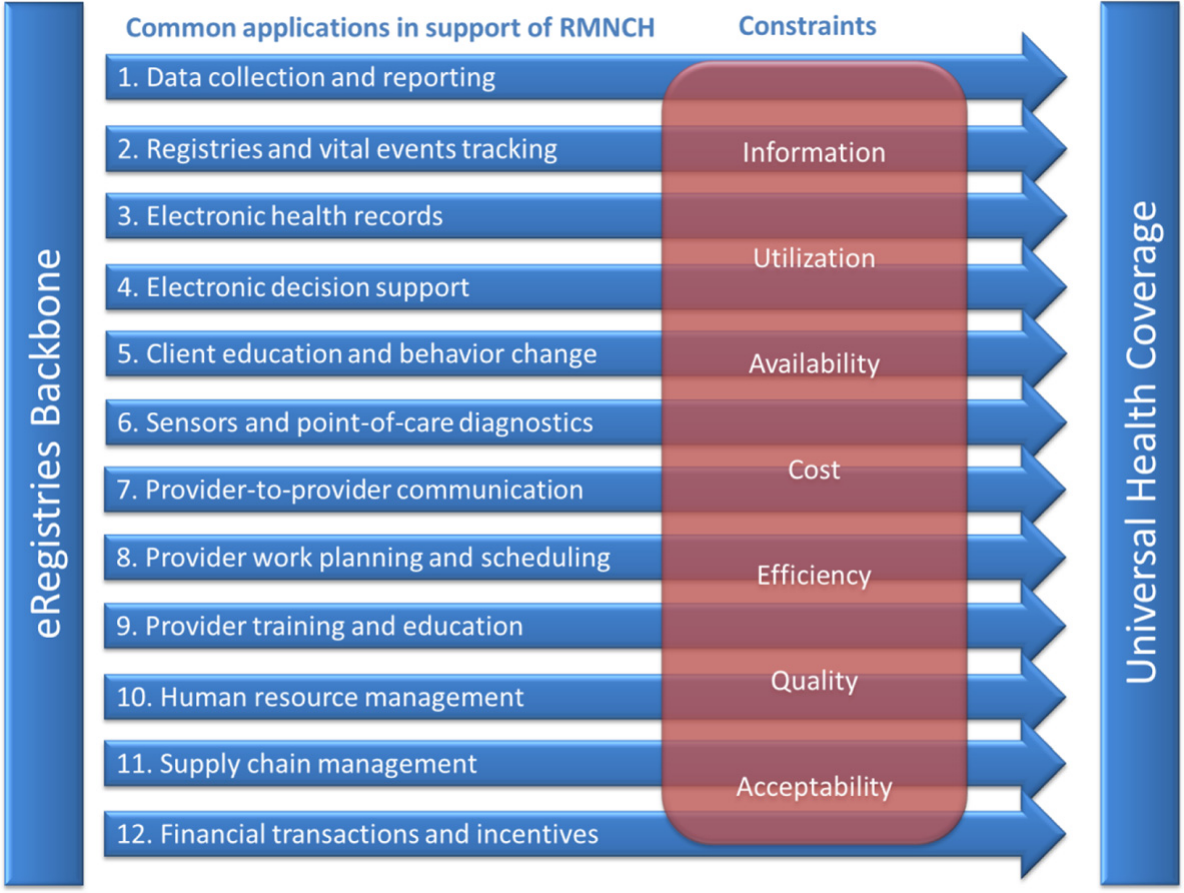
Framework for eRegistries in support of UHC in RMNCH. eRegistries in support of the 12 common electronic and mobile applications to overcome the seven principal constraints for universal health coverage in RMNCH, adapted from [24, 26]

The WHO RHR, the Johns Hopkins University Global mHealth Initiative, the United Nations Children’s Fund, and frog Design jointly developed the “mHealth and ICT [Information and Communication Technologies] Framework” to describe commonly used mHealth applications in RMNCH [24], and subsequently used for systematic review of evidence [25]. We used this framework and the taxonomy for primary health systems constraint categories developed by the WHO mHealth Technical and Evidence Review Group (mTERG) for RMNCH [26] to identify applications to alleviate common health systems constraints that can be supported by an eRegistry (Fig. 1).

### Survey of public health officials

Health officials working in any of the 75 LMIC monitored by the Commission on Information and Accountability for Women’s and Children’s Health, and Palestine, were recruited by emails to RMNCH medical and health organizations, ministries of health, public health institutes, and other relevant government offices (e.g., statistics bureaus, RMNCH departments, etc.). The survey was reviewed and provided with a Letter of Exemption by the Regional Committees for Medical and Health Research Ethics in Norway, confirming that the anonymous survey was not medical research on human subjects, and did not need ethical approval (Reference number: IRB 0000 1870). Launched in November 2013, responses were accepted until February 2015. The sample consisted of 298 individuals from 64 countries. Approximately two-thirds of respondents worked at the national or regional level. The survey included questions on national registry infrastructure, reporting and dissemination practices, and data quality. Issues such as privacy legislation, access by individuals and professionals, and data security are presented in the third paper, as well as additional details on the survey methodology (Myhre et al, submitted).

Country-level values are presented for all results. Decision rules for combining multiple responses into a single country response were adapted for each question; country averages were calculated for continuous data. Generalized linear models (PROC GLIMMIX) were used for confidence intervals around continuous data, while exact confidence intervals were calculated around binary and categorical data using SAS 9.4.

### Systematic search of literature

A systematic search of literature was conducted including papers from 2005–March 2015 using Medline, Embase, ISI Web of Science, Cochrane Library and Global Health. The searches used terms indicative of RMNCH registries and were limited to the 76 LMIC as above (Additional file 1). After de-duplication, 4237 articles were identified. We included studies based on longitudinal data collection systems for individual level RMNCH data, and excluded all alternative data collections such as cross sectional surveys and health record document reviews. Two investigators independently scored publications for inclusion and extracted data. We included the following data points (within the categories in parentheses): the country/-ies of operation, the extent of the registry data collection (in facilities only, in community services only, both, or not defined), the scale of the implementation of the registry (national, district, local, or not defined), the specified population captured by the registry data collection (a total population, only subgroups/select population, or not defined), the data collection method used (paper, electronic, both, or not defined), whether the primary data was collected and entered directly into the registry, or if the registry was based on a secondary/duplicate data collection from existing sources (direct, duplicated, or not defined). In cases of conflicting scores/data, consensus was reached after independent scoring by a third investigator. Full text was read for 302 publications with abstracts considered potentially relevant (Additional file 1).

## Results and discussion

The systematic and uniform data in eRegistries allows eHealth functionalities that give registries the potential to go far beyond simple registration tools [9, 27], and constitute an entire ecosystem of public health information and communication strategies (Frame 1). We mapped the potential uses of eRegistries in RMNCH onto two mHealth frameworks (Fig. 1): First, a set of 12 applications commonly used in RMNCH reflecting domains of work with empirical evidence of pervasive utility [24], and second, seven primary constraints to UHC for health systems (Frame 2) [26]:

**Frame 2:**
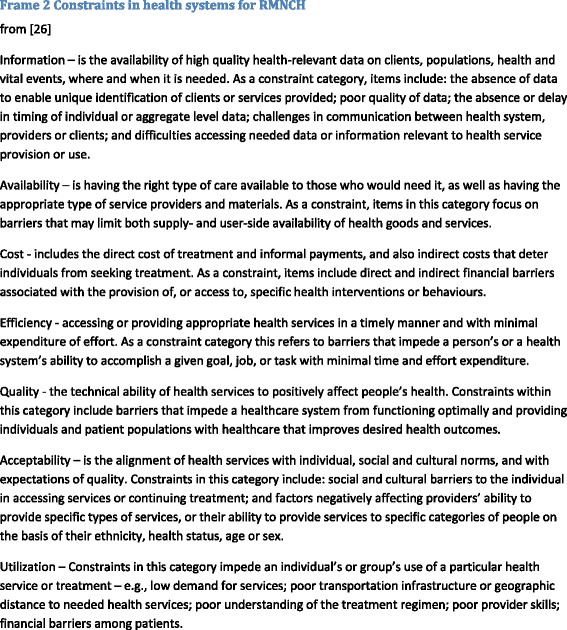
Constraints in health systems for RMNCH

### Commonly used mHealth applications within RMNCH

#### Data collection and reporting

Electronic HIS are in widespread use in LMIC to lessen health systems’ information constraints (Frame 2). Primary data creation in RMNCH occurs at the point-of-care, where frontline health workers document their clients’ health, services provided, and any specific data required for reporting to managers or national health statistics. A well-designed electronic application for systematic data collection and management will correspond intimately to the health workers’ needs in care provision [28, 29]. It can, at the same time, upload all or selected data items to create a registry for use in the care system. Additional electronically derived geographic information systems [30], and linkage of individuals to households [31, 32], can add to outreach utility and potentially link data to non-health data sources. Integrated with the national HIS, it allows managers to safely monitor and assess programs (Frost et al, submitted). This eliminates duplicate data entry for reporting which often consumes large proportion of provider’s time in LMIC [28, 33], easing constraints of health systems’ costs, availability and efficiency (Frame 2).

In registries in LMIC, real-time health surveillance tools are used for rapid quality improvement cycles [31]. LMIC registries contribute to independent prospective monitoring of trials and interventions [34–36], and large data collections are exploited to study rare conditions [35, 37, 38] and drug exposure [35, 36], or conversely, register rare conditions only [37, 39, 40]. Registries in LMIC follow mothers to study recurrence of pregnancy outcomes [41, 42], link mothers with offspring [29, 43–45] with their subsequent newborn health records [46], and link children with siblings [47–49]; in some cases registries study long-term and family-based health determinants and recurrence [41, 42] or undertaking twin studies [48, 49].

The ease of electronic feedback of data to providers is associated with improved quality of data [16]. eRegistry forms improve data quality with functionalities such as logical checks and limitations, warning prompts for improbable or missing data entries [29, 50, Frost et al, submitted], or pre-defined algorithms to improve correct categorizations, e.g. for causes of death [31]. Prospective and longitudinal data collection in eRegistries can reduce reporting bias on intervention coverage. For example, an adverse outcome can bias any retrospective registration of health determinants and care provision, i.e. what risks and health conditions she experienced in pregnancy and what was done to identify and manage them. This bias can make real-life estimations of effect of intervention coverage unhelpful.

#### Registries and vital events tracking

Recognizing the critical position of civil registration and vital statistics (CRVS) data in alleviating information constraints (Frame 2), the poor quality and coverage in LMIC, and the modest progress over the last 30 years, universal coverage of CRVS systems is a key goal for the post-2015 agenda [4, 51–53]. Well-functioning CRVS are independently associated with improved health, in particular lower child mortality [54]. Electronic and mobile applications are in widespread use for CRVS in LMIC. In settings with high proportions of home deliveries, mobile phones can extend the infrastructure by engaging frontline health workers to register births in communities [55, 56].

Being born is an essential health determinant, and dying likewise an essential outcome. CRVS systems thus share many characteristics and data with eRegistries for RMNCH that track individuals with unique identifiers (i.e. personal identification numbers (PIN)), or if lacking, issue such identifiers for care provision [57, 58]. Both in sentinel sites and total populations, several LMIC have created seamless integration of CRVS and HIS to benefit both enumeration of the population and support care provision, health monitoring, identify service delivery gaps and inequities, and improve accountability [38, 59]. Lack of such information can add to constraints of cost, acceptability and utilization of services by restricting eligibility (Frame 2).

The voluntary registration of all pregnancies can improve accountability and quality of vital events tracking related to RMNCH in LMIC [27, 60]. As pregnancy and date of birth are key in defining maternal mortality, eRegistries of pregnancies and births can facilitate correct classification when integrated with a CRVS registering deaths of women in fertile age. Stillbirths and neonatal deaths are poorly registered in many settings with a high percentage of home deliveries, stigma, and a lack of incentives to register a dead baby, for either care professionals, or for parents. The magnitude of the issue can be unmasked by counting third trimester pregnancies registered in eRegistries with no subsequent report of a live infant, civil registration, vaccination or other incentivizing benefits that can be integrated or linked to the registry.

#### Electronic health records

As stated in MA4Health, quality, cost and efficiency constraints cannot be overcome without patient data being shared across sites and levels of care (Frame 2) [5]. RMNCH has a long tradition of using simplified client-held paper records of systematic and uniform data for communication along the continuity of care and to facilitate self-care [61]. This tradition makes RMNCH particularly fitting for the use of eRegistries where systematic and uniform data are the key to functionality, including the ability to manage data items such as unstructured text notes and narratives as in health records. Conversely, electronic health records built on an architecture of unstructured text and forms as data items can not support the functionalities of an eRegistry. In LMIC, the traditional maternity, child health and vaccination cards are valued, and while loss of records by women is not typically reported as a major problem, the communication flow can be broken as they are often not brought to care visits, and confidentiality has been questioned [61]. The value of information following the woman is evident in societies with traditions wherein women travel to their parental home to deliver. In such settings, single facility based or local electronic systems can not only be expensive and hard to maintain [62], but represents an inferior tool for information management – although some projects have provided women with printed versions [32] or uploaded records to a server for women themselves to have electronic access [58].

Even in settings of less mobility in care seeking, a backbone registry accessible across its regional or national jurisdiction supports continuity of health records at all levels of care, with secure governance and storage preventing irreversible loss or damage to facility or client-held paper records. An electronic version adds to patient safety by also making information available in emergencies when a paper card may be unavailable. Mobile units enable entries and access to health records from community and outreach activities. In LMIC, research based on registries often extract their data from electronic health records (Additional file 1). Some also link individuals’ records to biobanks and lab tests [49].

#### Electronic decision support

Best practice guidelines are well-established in RMNCH, and achieving effective coverage of interventions, i.e. high quality of care, is key in the post-2015 agenda [63–65]. Guidelines may appear straightforward, but are seldom followed in the correct and complete sequence. This know-do gap constrains quality of care services (Frame 2) [66, 67]. A commonly cited hindering factor is unavailability of guidelines in a user-friendly, readily accessible manner at the point-of-care [68, 69].

Checklists are informational job aids extracting vital elements of guidelines for clinical care to simplify the presentation and highlight actions required. They are in common use to reduce variation in performance and assist in improving quality of care in LMIC [69–71]. Corresponding intimately to the systematic and uniform approach to data in eRegistries, data entry at the point-of-care in eRegistries can be designed as interactive checklists, which integrate decision support for diagnosis, treatment and referral algorithms (Frame 1) [32, 69, Frost et al, submitted].

Integrated decision support and reminders for guideline adherence in preventive care are shown to strengthen health systems and link HIS to improved quality coverage of care [72–75]. Such tools are generally associated with high user satisfaction, but require training for use, and developing comprehensive tools for all essential interventions and guidelines in RMNCH requires substantial preparations to ensure that they reflect the actual work flow of providers [69, 76, 77, Frost et al, submitted]. However, most studies exploring these effects have been conducted in facility settings, and better evidence is needed to enhance community based RMNCH care in LMIC [78–80].

#### Client education and behavior change communication

Feeding back the registered information to individuals contributes by informing the public about the objectives and values of registration, as the UN recommends for CRVS. But the underutilization of data to empower women and communities has largely been overlooked. The use of registry data for community and client education and behavior change communication, has the potential to impact utilization and acceptability constraints, as well as empower women to demand improvements in health system quality, accessibility, cost and efficiency (Frame 2).

Women are principal stakeholders for their own information, and communicating it back to them should be personalized, timely and actionable. General pregnancy information can be of variable interest to the individual woman if not tailored to her needs, and mHealth solutions to communicate with her may not help much unless personalized [77]. Therefore, efforts have been made in LMIC to register women and children to deliver pregnancy stage and age appropriate messaging [81–83]. mHealth solutions building on eRegistries can communicate to her mobile phone or web-applications with personalized and culturally sensitive information according to the data registered about her. eRegistries can automate provision of information directly to women, or as a prompt to her care provider to send the information, to complement in-person approaches and assist in bridging communication barriers with multi-language support for messaging. For example, gestational age data can ensure timely advice for birth preparation, while risk or complication data can tailor information for high risk pregnancies, services or treatment data can prompt reminders of medications or appointments, and vaccination data and residential address can be used to inform of available outreach vaccination services. Such information on the importance and availability of services can empower the demand for services and improve health care utilization and acceptability (Frame 2). One of the most established applications of mHealth in LMIC is messaging of reminders for appointments and treatments, to improve care utilization and efficiency [80].

Crucially, reliance on mobile phone access for communication frequently raises questions about equity [25]. Not having access to a mobile phone is a significant marker of risk for poor outcomes that should be registered, and alternative communication methods should be provided [84].

#### Sensors and point-of-care diagnostics

The restriction of diagnostics to fixed site laboratories adds constraints of availability, cost, acceptability and utilization (Frame 2). Miniaturized point-of-care diagnostic tests and sensors combined with the computing, storage and communication power of mobile phones and tablets have led to a rapidly expanding range of mHealth innovations for diagnostic tests in communities [85]. Standard tests in RMNCH from blood and urine, and external sensors for fetal Doppler and blood pressure exist in low-cost mobile units for LMIC settings. The results of such tests is key information both for RMNCH care provision and health surveillance, and should be integrated in the backbone HIS. An example of such successful integration in LMIC is the implementation of Swasthya Slate, linking a small independent diagnostic unit to a tablet used by frontline community health workers to upload individual results to a cloud-based eRegistry available to care professionals, clients, and program managers [58].

#### Provider-to-provider communication

Insufficient communication at hand-offs and referrals significantly constrains quality and efficiency (Frame 2). An integrating backbone HIS for seamless information-sharing across multiple providers and levels of care is a key element in MA4Health’s global roadmap. Where disconnected cadres of providers interact with the same client, duplicating care, information and reporting efforts, shared health records implicitly constitute a key type of provider-to-provider communication. Within functioning health systems, shared information can cut delays and time spent in hand-offs and referrals to other providers—whether simplifying a traditional referral, or in the simplest form reduced to an automated transfer from one provider’s electronic work schedule to another’s. Mobile solutions can extend the reach of real-time information sharing e.g. of results from laboratories to frontline health workers.

Paper records are not only logistically difficult to share, but are reputed for illegibility and incompleteness [28]. From a client-held antenatal care paper record, it can be difficult to identify the care providers managing a woman, hampering their ability to communicate. For providers to be accredited as users in eRegistries, a unique user identification is created, and every entry of information logged to the individual provider (Frost et al, submitted). Their contact information can be available to others engaged in providing services to the client, and to the clients themselves, facilitating communication and identifying unwarranted “shopping” of services across providers.

#### Provider work planning and scheduling

Shortage of care providers in LMIC severely constrains availability, quality and efficiency of RMNCH services (Frame 2). Resources are wasted not only by underutilizing the time-saving benefits of electronic applications discussed here, but also by inefficiencies such as clients missing scheduled appointments or unneeded variation in daily work-load [80]. Likewise, systems become inefficient in providing adequate care when they are unaware of the time and location at which clients will require care. Optimizing the efficiency of the work force has therefore been a key element in many deployments of e- and mHealth programs in LMIC, including functionalities ranging from simple electronic scheduling, or household visit support with geographic information systems, to integration of messaging services to create mobile phone reminders about upcoming antenatal care visits, missed scheduled appointments, new deliveries and newborns eligible for postpartum and newborn care [31, 32, 86].

#### Provider training and education

Poorly performing providers cause quality, cost, efficiency, utilization and acceptability constraints (Frame 2). Interactive mHealth solutions are used for continued medical education and training support in LMIC—mostly in generic forms applied to a cadre of health workers. A standard course may not be professionally motivating for providers at different levels of performance. eRegistries create inherent accountability with data on the client population, care performance and outcomes, which facilitate individually targeted training, including the potential for automated audit and feedback [69, 87].

The variability in the approaches and results of audit and feedback interventions, including those specifically using registry data, might be explained by limited consensus and use of theories underlying multiple causal pathways [88–93]. Reviews in the framework of Feedback Intervention Theory have identified that verbal, discouraging, praising or self-esteem-affecting feedback attenuates beneficial effects on performance, while task-oriented computerized feedback augments it [94, 95]. Frequent, swift, and correct solution feedback, including a goal setting action plan, also augment effects. Effects are stronger for familiar memory tasks and weaker for following rules and completing complex tasks [94, 95]. The Model of Actionable Feedback further proposes that in order to be actionable, feedback must be timely, individualized, non-punitive and meaningful [96, 97]. These findings fit well with systematic reviews of feedback to healthcare providers that also identified larger effects if it was delivered to non-physicians by a supervisor or trustworthy colleague, in a domain where the recipient was underperforming [91, 92].

There is evident potential of eRegistries to deliver computerized, individualized, trustworthy, non-punitively neutral, timely, frequent and task-oriented feedback to health workers to focus attention to the largest quality gaps. However, stronger evidence is needed. Few studies have evaluated the effect of feedback on improving RMNCH, and studies from LMIC are lacking [91, 92, 98].

#### Human resource management

Closely linked to applications for work planning and scheduling, as well as those improving working conditions and satisfaction, better human resource management is needed to meet workforce constraints. Population-based data on health, health determinants and effective coverage of services is necessary for better and more targeted distribution of health workers. This is a critical issue in many LMIC settings where extremely low provider to client ratios remain a long-term problem. Monitoring and management of public health providers in LMIC is made more complex by prevalent dual practice—individual professionals providing services both privately and in the public health system—which in poorly regulated settings can add constraints to accessibility, cost, efficiency, quality, as well as equity (Frame 2) [99]. Information on actual performance of the workforce is therefore needed. As providers accessing individual patient data have unique user identities, eRegistries also represent provider registries, where their service provision is logged. Just as client data can be aggregated or disaggregated from national to individual level, so can provider data, joined with client data to support allocation of human resources needed to deliver services based on the number of beneficiaries [32]. Supportive supervision can enable program managers to identify lower quality of services than expected given the resources invested, and the real-time registration of services provided, can contribute in addressing absenteeism and enabling substitution when critical services are not provided.

#### Supply chain management

mHealth applications in Logistics Management Information Systems (LMIS) are widespread in LMIC to improve data visibility, enhance decision making, and address availability and cost constraints of health systems (Frame 2) [100]. Paper LMIS have low reporting rates in LMIC, due to e.g. unreliable postal systems, poor transport infrastructure, and a heavy workload among store managers. Electronic and mobile LMIS address these constraints, improving reporting rates, data visibility, and use [101].

eRegistries contain key data to combine with LMIS for improved quantification, forecasting and distribution of health commodities, including dispensed-to-users data, provider and client needs and preferences, and seasonality. The rich patient data available from eRegistries can also improve prediction of future needs, using better-quality information about population risk and demographics. Integration of HIS and LMIS is rarely seen [32], typically operating as isolated silos, but is recommended by the UN Commission on Life-Saving Commodities for Women and Children [102]. Combining eRegistry and LMIS data can provide another tool for transparency and accountability, comparing numbers of commodities issued from storage with those dispensed to users to identify potential theft/leakage.

#### Financial transactions and incentives

The use of mobile payments in LMIC bring financial services within reach of previously unbanked populations. Throughout LMIC, 60 % now have coverage of mobile financial services, and sub-Saharan Africa is leading other regions in the number of deployments [103]. This can assist in alleviating accessibility, acceptability and utilization constraints (Frame 2), and eRegistries in RMNCH can facilitate the use of such transactions and incentives. This includes making information available at point-of-care on eligibility for universal health insurance schemes, data on service delivery for performance based incentives, on registered conditions eligible for financial support such as transportation for institutional delivery, or incentives for child vaccination.

### Country capacity for electronic health registries in RMNCH

While the potential of eRegistries are clear, the perceived needs and capacities in LMIC to deploy them are not. Unique identifier systems issuing PINs are a requirement for any population registry, enabling detection and elimination of duplicate records, or fraudulent identities. It also enables linkages through integrated backbone systems for multiple data entries and uses as in eRegistries.

According to the public health officials surveyed, 60 % (95 % confidence interval: 47–72) of LMIC issue PINs for both permanent and temporary residents while 17 % (9–29) of countries issue them to permanent residents only. A small percent (3 % (0–11)) indicated that the system was in an initial stage and one sixth indicated that they did not issue PINs; three percent did not know. Eight out of ten LMIC indicating that they do not issue PINs were located in the African region. In the vast majority of countries with PINs, the use was reported to be proof of citizenship (94 % (84–99)), or needed for access to education (57 % (42–71)), health services (51 % (36–65)), financial services (53 % (38–67)), and for taxation (45 % (31–60)). Few offer automatic registration at birth. Barriers to birth registration indicate that home delivery, cultural mores, societal taboos, and religious traditions all contribute to low registration practices at birth [104]. Adult applications are the most common method for acquiring a PIN. Half of countries offer only one option to obtain a PIN whereas 51 % (37–65) offer multiple options (Table 1).

**Table 1:** Unique identity systems’ administrative oversight and acquisition

Agency in charge	Frequency	Percent^a^
Ministry of Health	7	14 % (6–26)
All other Ministry offices	41	80 % (67–90)
National office/agencies	34	67 % (52–79)
Local level offices/agencies	13	25 % (14–40)
Acquisition		
Application required at birth	27	53 % (38–67)
Automatically issued at birth	11	22 % (11–35)
Application required as an adult^b^	30	59 % (44–72)
Automatically issued as an adult^b^	6	12 % (4–24)
Other	8	16 % (7–29)

^a^Percent column does not add up to 100 % as the question asked respondents to check all that apply

^b^Adult was considered 16 years or older. 95 % confidence intervals in parentheses

The latest 2013 UNICEF report on birth registration, based on informants from household surveys with data on average from 2010, reported that about four in ten children under five were registered in CRVS in LMIC. A higher proportion of countries report in our survey that they do at least register births. Nearly half of countries report coverage greater than 90 % for registering birth, pregnancy and child health, but many regions still experience very low coverage, and a recorded birth does not translate to formal birth registration to acquire a PIN. Closer integration with birth registration in health systems may facilitate the registration process [104]. Cause of death registries are of notoriously poor coverage and quality in many regions (Table 2) [105].

**Table 2:** Coverage of vital and health statistics in national registries

Registry (Countries)	>90 % coverage	50–89 % coverage	<50 % coverage	I don’t know	No national registry
Birth (*N* = 62)	55 % (42–68)	27 % (17–40)	10 % (4–20)	6 % (2–16)	2 % (0–9)
Cause of death (*N* = 61)	38 % (26–51)	21 % (12–34)	20 % (11–32)	16 % (8–28)	5 % (1–14)
Pregnancy (*N* = 61)	43 % (30–56)	34 % (23–48)	11 % (5–22)	10 % (4–20)	2 % (0–9)
Child (*N* = 62)	45 % (32–58)	26 % (16–39)	15 % (7–26)	11 % (5–22)	3 % (0–11)

95 % confidence intervals in parentheses

Overall, the responding public health officials in LMIC rate the quality of their national health data quite modestly on a scale from 5 (excellent) to 1 (poor): A quarter of countries have health data scored as poor by their officials in terms of timeliness and security, one fifth as poor on completeness and update status, and across all domains of data quality (accuracy, completeness, integrity, access, security, timeliness, update status and utilization) average scores range from 2.3 to 2.5 ((95 % confidence interval: 2.1–2.6). Nonetheless, on a range from 5 (very influential) to 1 (not influential at all), public health officials generally score the influence of national data reports on public health decision-making highly. Data had the highest influence on planning public health programs and monitoring of the Millennium Development Goals (average score 4.3 (4.1–4.4)), somewhat less on monitoring, evaluation and improvement of health programs (3.9–4.1 (3.6–4.3)), and the least influence on budget allocations (3.4 (3.2–3.6)) and research (3.7 (3.4–3.9)). For other use of data for feedback to the health system, respondents generally score the quality as low as the quality of the data (same scale): The highest quality of feedback for effective coverage of antenatal care (2.7 (2.5–2.9)), and the lowest for quality of care in facilities (2.3 (2.1–2.5)).

Most LMICs are in a phase of transition from paper to mixed paper and electronic formats, and only a low percentage report using fully electronic registries (Table 3). With the tardiness of paper registrations, approximately half the countries have mortality figures updated annually, while one in five report a time lag from data collection to publicly available reports of more than three years on average. Data on intervention coverage in antenatal, delivery, postpartum and newborn care collected by health professionals fare similarly with two of three countries reporting having this publicly available within one year of data collection.

**Table 3:** Data transmission format from health facilities to central data collection for registries

Registry (Countries)	Electronic	Paper & Electronic	Paper	No national registry	I don’t know
Birth (*N* = 62)	5 % (1–14)	69 % (56–80)	18 % (9–30)	3 % (0–11)	5 % (1–14)
Cause of death (*N* = 59)	3 % (0–12)	61 % (47–73)	19 % (10–31)	7 % (2–16)	10 % (4–21)
Pregnancy (*N* = 61)	3 % (0–11)	61 % (47–73)	25 % (15–37)	5 % (1–14)	7 % (2–16)
Child (*N* = 61)	3 % (0–11)	66 % (52–77)	18 % (9–30)	7 % (2–16)	7 % (2–16)

95 % confidence intervals in parentheses

### Scientific capacity for electronic health registries in RMNCH

Capacity to operate eRegistries of high scientific quality in LMIC should be expected to be an important constraint given the slow emergence of such registries and the current status of data. From 2005 to 2015, we identified 66 publications from 32 health registries in 24 LMIC—from a broad search in LMIC literature in the field of RMNCH resulting in 4237 abstracts screened and 302 papers read in full. The registries identified are presented in Table 4.

**Table 4:** RMNCH Registries with scientific publications

Countries	Registry scope	Registry scale	Population	Data collection format	Data entry source	Health focus^a^	Registry name and operation^b^
Brazil	Community	National	Total	E & Paper	Duplicate	A + CRVS	Brazilian National Birth Registry (SINASC) - N
Peru	Facility	National	Total	Electronic	Primary	B + CRVS	Online Registration of Certificates of Live Births - N
China	Facility	National	Sample	E & Paper	Duplicate	B + Birth defects	Birth Defects Monitoring Network - I
Egypt	Community	National	Total	E & Paper	Duplicate	A + IVF	Egyptian IVF registry - N
Peru	Community	National	N/A	Electronic	Primary	ABC	Peruvian Perinatal Information System (SIP) -I
Ghana	Both C & F	Regional	Total	E & Paper	Duplicate	ABCD	Navrongo Health and Demographic Surveillance System-NI
Kenya	Community	Regional	Total	Electronic	Primary	ABD	KEMRI Centers for Disease Control & Prevention-NI
Yemen	Community	Regional	Total	Electronic	Duplicate	C + Cancer	Aden Cancer Registry- N
Kenya	Community	Regional	Total	E & Paper	Duplicate	ABD + Vaccines	Kilifi Health and Demographic Surveillance System -NI
Uganda	Community	Regional	Total	E & Paper	Duplicate	A + HDR	Health and Demographic Surveillance Site (HDSS) -NI
Guinea-Bissau	Facility	Regional	Total	E & Paper	Duplicate	AC + Twins, Vacc.	Bandim Health Project (BHP) - NI
Guinea-Bissau	Facility	Regional	Total	E & Paper	Duplicate	AC + Twins, Vacc.	Guinea-Bissau Twin Registry-NI
China	Community	Regional	Sample	Electronic	Primary	C + Vaccines	Zhejiang Immunization Information System (ZJIIS) - N
Bangladesh	Community	Regional	Sample	Paper	Duplicate	AB	A registry for the JiVitA 1 project - NI
Zambia	Facility	Regional	Sample	Electronic	Primary	AB	Zambia Electronic Perinatal Record System NI
Burkina Faso, Ghana, Tanzania	Facility	Regional	Sample	Electronic	Primary	AB	Quality of Prenatal & Maternal Care Clinical Decision Support (QUALMAT) - NI
Tanzania	Facility	Regional	Sample	Paper	Duplicate	ABC	KCMC Medical Birth Registry - NI
Chile	Facility	Regional	Sample	Electronic	Duplicate	A + ART	Latin American Registry (RLA) of assisted reprod. tech. - NI
Senegal	Community	Regional	Sample	E & Paper	Duplicate	ABD	Millennium Villages Project information system- NI
Indonesia	Community	Local	Total	E & Paper	Duplicate	ABC + Malaria	Sistem Informasi Kesehatan Daerah (SIKDA)- NI
China	Community	Local	Total	E & Paper	Duplicate	ABC	Gongcheng Maternal, Newborn and Child Health Information System - NI
Bangladesh	Both C & F	Local	Sample	E & Paper	Duplicate	A	Projahnmo-II Project - Pregnancy Registration - NI
Kenya, Pakist., Guatem., Zambia, India, Argent.	Both C & F	Local	Sample	E & Paper	Duplicate	ABC	Global Network Maternal and Newborn Health Registry - NI
Nigeria	Facility	Local	Sample	E & Paper	Duplicate	AC + Vaccines	OpenMRS for the Family Health Unit - NI
Kenya	Facility	Local	Sample	E & Paper	Duplicate	AB + HIV	Academic Model Providing Access to Healthcare (AMPATH)- NI
Cameroon	Facility	Local	Sample	E & Paper	Duplicate	AB	Obstetric health information system -NI
Nepal	Facility	Local	Sample	E & Paper	Primary	A + CVD	Prospective single-centre registry - NI
Brazil, Ghana, Kenya, Uganda, Tanzania	Community	Local	Total	E & Paper	Duplicate	A + Drug expo.	WHO Pregnancy Registry of drug exposures using OpenClinica - NI
Brazil	Facility	Local	Total	E & Paper	Primary	B + Vaccines	Quality-Health Card/Electronic Immunisation Registry-N
India	Facility	Local	Sample	E & Paper	Duplicate	ABC + Epilepsy	Indian Registry of Epilepsy and Pregnancy (IREP) -N

^a^ *A* Pregnancy, *B* Childbirth, *C* Infant/child, *D* Deaths

^b^ *N* Operated nationally, *I* Operated by international organizations, *NI* Operated in collaboration between national and international organizations. *N/A* Not available information in publications. References in Additional file 1

The majority of health registries in RMNCH that have reached the mature stage of authoring scientific publications, operate at a scaled regional or national level, and they have come further in the transition from paper to electronic data capture than the national registration systems reported from national levels in the survey. Countries such as Kenya, Zambia, Burkina Faso, Ghana and Tanzania have all published research based on primary data entry in fully electronic registries at national or regional levels. While data collection format and the source of primary data is often poorly described in publications, it appears that in most countries, undertaking research in registry methodology still represents duplicate data collection efforts to transfer data from paper forms to an electronic database.

## Conclusions

We find that purposefully designed eRegistries, acting as an integrating backbone HIS, can be the operative infrastructure for several commonly deployed applications to strengthen RMNCH, and offer crucial data and support for all. Collectively, they have the potential to alleviate the principal constraints of health systems for UHC in RMNCH. Yet, although many examples exist of successful implementation of individual applications based on registry functionalities, none have integrated more than a few within one single backbone system. While electronic solutions with registry functionalities are widely used in LMIC for data collection, reporting, CRVS, health records and clinical decision support, planning and scheduling, and inherently for communication of health information between providers, they appear underutilized for client behavior change communication, provider audit and feedback, and management of human resources, supply chains and financial incentives.

Almost all countries are investing resources into systems to support registration of births and vital events in RMNCH, and the majority are currently in transition from paper to a future of electronic HIS where eRegistries could become an integrating backbone and contribute to relieve the constraints they experience in data collection, management, analysis and dissemination. Public health officials in LMICs convey a strong message of the importance their national RMNCH-related data for country policies and program management, despite the many times mediocre coverage and quality, and long delays and suboptimal quality of feedback of data to the health system. The MA4Health post-2015 roadmap and action plan should further galvanize national investment and commitments to support the transition to electronic solutions [4, 106].

There is accumulating experience on operating health registries in LMIC to draw lessons from, and eRegistries are emerging at regional and national scale. While the finding for infrastructure capacities is encouraging for future emergence of eRegistries in RMNCH, the scientific activity is limited.

Capacity building and support for eRegistries is crucial to achieve the goals set for the post-2015 agenda reflected in the MA4Health roadmap. In this series, we offer tools and frameworks to achieve that end, including key considerations in the selection of data items and indicators, architectural standards and interoperability, and ethics and governance.

## Additional file

Additional file 1:
**Systematic review methodology supplementing information.** (DOCX 141 kb)

## Abbreviations

CRVS: civil registration and vital statistics
eHealth: (See Frame 1)
eRegistries: (See Frame 1)
HIS: Health information systems
LMIC: Low- and middle-income countries
LMIS: Logistics management information systems
MA4Health: World Bank/WHO/USAID measurement and accountability for results in health: a common agenda for the post 2015 era
mHealth: Mobile eHealth (See Frame 1)
PIN: Personal identification numbers
RMNCH: Reproductive, maternal, newborn and child health
UHC: Universal health coverage
UN Global Strategy: United Nations: Survive, Thrive, Transform. The Global Strategy for Women’s, Children’s and Adolescents’ Health
WHO: World Health Organization
