# Supplementary material for: eRegistries: Electronic registries for maternal and child health
Source: BMC Pregnancy Childbirth. 2016 Jan 19;16:11. doi: 10.1186/s12884-016-0801-7 (PMC4721069; doi:10.1186/s12884-016-0801-7)
Supplement: Additional file 1: — Systematic review methodology supplementing information. (DOCX 141 kb) [file 12884_2016_801_MOESM1_ESM.docx]

# Systematic review methodology supplementing information

### Search Terms

The following are the search terms used for Medline, Embase, ISI Web of Science, Cochrane Library and Global Health. The search terms were inclusive of all 75 Commission on Information and Accountability for Women's and Children's Health (CoIA) countries and Palestine. The searches used terms indicative of reproductive, maternal, newborn and child health (RMNCH) registries and limited to the 75 CoIA countries and Palestine. The search terms included the following -:

### MEDLINE, EMBASE and Global Health

1. registries/

2. registries.mp.

3. registry.mp.

4. register/

5. register.mp.

6. birth registr*.mp.

7. medical record/

8. medical record*.mp.

9. health record*.mp.

10. public health informatics/

11. public health informati*.mp.

12. medical informatics/

13. medical informatic*.mp.

14. "forms and records control"/

15. "forms and records control".mp.

16. "reproducibility of results"/

17. "reproducibility of results".mp.

18. "reproducibility of findings".mp.

19. OR/1-18

20. reproductive health/

21. reproductive health*.mp.

22. maternal welfare/

23. maternal welfare*.mp.

24. maternal health*.mp.

25. maternal child health*.mp.

26. infant, newborn/

27. newborn infant*.mp.

28. newborn*.mp.

29. newborn/

30. pregnancy/

31. pregnan*.mp.

32. parturition/

33. parturition.mp.

34. childbirth*.mp.

35. birth/

36. birth*.mp.

37. OR/20-36

38. 19 and 37

39. angola.mp.

40. benin.mp.

41. botswana.mp.

42. burkina faso.mp.

43. burundi.mp.

44. cameroon.mp.

45. central african republic.mp.

46. chad.mp.

47. comoros.mp.

48. congo.mp.

49. ivory coast.mp.

50. cote d'ivoire.mp.

51. Democratic Republic of Congo.mp.

52. equatorial guinea.mp.

53. ethiopia.mp.

54. eritrea.mp.

55. gabon.mp.

56. gambia.mp.

57. ghana.mp.

58. guinea.mp.

59. guinea-bissau.mp.

60. kenya.mp.

61. lesotho.mp.

62. liberia.mp.

63. madagascar.mp.

64. malawi.mp.

65. mali.mp.

66. mauritania.mp.

67. mozambique.mp.

68. niger.mp.

69. nigeria.mp.

70. rwanda.mp.

71. sao tome.mp.

72. senegal.mp.

73. sierra leone.mp.

74. south Africa.mp.

75. swaziland.mp.

76. togo.mp.

77. uganda.mp.

78. tanzania.mp.

79. zambia.mp.

80. zimbabwe.mp.

81. bolivia.mp.

82. brazil.mp.

83. guatemala.mp.

84. haiti.mp.

85. mexico.mp.

86. peru.mp.

87. afghanistan.mp.

88. djibouti.mp.

89. egypt.mp.

90. iraq.mp.

91. morocco.mp.

92. pakistan.mp.

93. somalia.mp.

94. south sudan.mp.

95. sudan.mp.

96. yemen.mp.

97. azerbaijan.mp.

98. (Kyrgyzstan OR Kirghizstan OR Kirgizstan).mp.

99. (Tajikistan OR Tadzhikistan OR Tadjikistan).mp.

100. Turkmenistan.mp.

101. Uzbekistan.mp.

102. bangladesh.mp.

103. "D.P.R. Korea".mp.

104. "democratic people's republic of korea".mp.

105. DPRK.mp.

106. north korea.mp.

107. india.mp.

108. indonesia.mp.

109. myanmar.mp.

110. burma.mp.

111. nepal.mp.

112. cambodia.mp.

113. Kampuchea.mp.

114. china.mp.

115. laos.mp.

116. "lao P.D.R.".mp.

117. "Lao People's Democratic Republic".mp.

118. papua new guinea.mp.

119. philippines.mp.

120. solomon islands.mp.

121. (viet nam OR vietnam).mp.

122. OR/39-121

123. 38 and 122

124. remove duplicates from 123

125. conference abstract.pt.

126. letter.pt.

127. editorial.pt.

128. OR/125-127

129. 124 not 128

### ISI Web of Science

TS=(registries OR registry OR register OR"birth registr*" OR "medical record*" OR "health record*" ORr "public health informati*" OR "medical informatic*" OR "records control" OR "reproducibility of results" OR "reproducibility of findings")

AND

TS=("reproductive health*" OR"maternal welfare*" OR "maternal health" OR "maternal child health" OR "newborn infant*" newborn* OR pregnan* OR parturition OR childbirth* OR birth)

AND

TS=(angola OR benin OR botswana OR "burkina faso" OR burundi OR cameroon OR "central african republic" OR chad OR comoros OR congo OR "ivory coast" OR "cote d'ivoire" OR "Democratic Republic of Congo" OR "equatorial guinea" OR ethiopia OR eritrea OR gabon OR gambia OR ghana OR guinea OR "guinea-bissau" OR kenya OR lesotho OR liberia OR madagascar OR malawi OR mali OR mauritania pr mozambique OR niger OR nigeria OR rwanda OR "sao tome" OR senegal OR "sierra leone" OR "south africa*" OR swaziland OR togo OR uganda OR tanzania OR zambia OR zimbabwe OR bolivia OR brazil OR guatemala OR haiti OR mexico OR peru OR afghanistan OR djibouti OR egypt OR iraq OR morocco OR pakistan OR somalia OR "south sudan" OR sudan OR yemen OR azerbaijan OR Kyrgyzstan OR Kirghizstan OR Kirgizstan OR Tajikistan OR Tadzhikistan OR Tadjikistan OR Turkmenistan OR Uzbekistan OR bangladesh OR "D.P.R. Korea" OR "democratic people's republic of korea" OR DPRK OR "north korea" OR india OR indonesia OR myanmar OR burma OR nepal OR cambodia OR Kampuchea OR china OR laos OR "lao P.D.R." OR "lao PDR" OR "Lao People's Democratic Republic" OR "papua new guinea" OR philippines OR "solomon islands" OR "viet nam" OR Vietnam OR palestine)

### Cochrane Library

(registries OR registry OR register OR “birth registr*” OR “medical record*” OR “health record*” OR “public health informati*” OR “medical informatic*” OR “records control” OR reproducibility)

AND

("reproductive health*" OR "maternal welfare*" OR "maternal health" OR "maternal child health" OR "newborn infant*" newborn* OR pregnan* OR parturition OR childbirth* OR birth)

AND

(angola OR benin OR botswana OR "burkina faso" OR burundi OR cameroon OR "central african republic" OR chad OR comoros OR congo OR "ivory coast" OR "cote d'ivoire" OR "Democratic Republic of Congo" OR "equatorial guinea" OR ethiopia OR eritrea OR gabon OR gambia OR ghana OR guinea OR "guinea-bissau" OR kenya OR lesotho OR liberia OR madagascar OR malawi OR mali OR mauritania OR mozambique OR niger OR nigeria OR rwanda OR "sao tome" OR senegal OR "sierra leone" OR "south africa*" OR swaziland OR togo OR uganda OR tanzania OR zambia OR zimbabwe OR bolivia OR brazil OR guatemala OR haiti OR mexico OR peru OR afghanistan OR djibouti OR egypt OR iraq OR morocco OR pakistan OR somalia OR "south sudan" OR sudan OR yemen OR azerbaijan OR Kyrgyzstan OR Kirghizstan OR Kirgizstan OR Tajikistan OR Tadzhikistan OR Tadjikistan OR Turkmenistan OR Uzbekistan OR bangladesh OR "D.P.R. Korea" OR "democratic people's republic of korea" OR DPRK OR "north korea" OR india OR indonesia OR myanmar OR burma OR nepal OR cambodia OR Kampuchea OR china OR laos OR "lao P.D.R." OR "lao PDR" OR "Lao People's Democratic Republic" OR "papua new guinea" OR philippines OR "solomon islands" OR "viet nam" OR Vietnam OR palestine)

### Flow Diagram

**Data Base Search**

Medline; EMBASE; ISI Web of Science; Cochrane Library and Global Health

**Total N = 4778**

**Total n = 4778**

## Identification

Abstracts excluded after screening

**Total N = 3935/4237**

Abstracts included after screening

**Total N = 302/4237**

Included articles for data synthesis

**Total N = 43/66**

Records after duplicates removed

**Total N = 4237**

Eligible full-text articles selected
**Total N =66/302**

## Screening

## Eligibility

## Included

### References related to systematic review

### Below is a list of references according to the systematic review findings presented in the paper in Table 4.

Brazil - [1, 2]

Peru - [3]

China - [4]

Egypt - [5]

Peru - [6]

Ghana - [7]

Kenya - [8]

Yemen - [9]

Kenya - [10]

Uganda - [11]

Guinea-Bissau - [12]

Guinea-Bissau - [13]

China-[14]

Bangladesh - [15]

Zambia - [16]

Burkina Faso, Ghana, Tanzania[17]

Tanzania - [18-27]

Chile- [28]

Senegal - [29]

Indonesia - [30]

China - [31]

Bangladesh - [32]

Kenya, Pakistan, Guatemala, Zambia, India and Argentina - [33-36]

Nigeria - [37]

Kenya - [38]

Cameroon - [39]

Nepal - [40]

Brazil, Ghana, Kenya, Uganda and Tanzania - [41]

Brazil - [42]

India - [43]

1. Nhoncanse GC, Melo DG: **Reliability of birth certificates as a source of information on congenital defects in the City of Sao Carlos, Sao Paulo, Brazil**. *Ciencia & Saude Coletiva* 2012, **17**(4):955-963.

2. Guimaraes PV, Coeli CM, Cardoso RCA, Medronho RdA, Fonseca SC, Pinheiro RS: **Reliability of data from a very low birth weight population in the Live Birth Information System 2005-2006**. *Revista Brasileira de Epidemiologia* 2012, **15**(4):694-704.

3. Curioso WH, Pardo K, Loayza M: **Transforming the peruvian birth information system Transformando el sistema de informacion de nacimientos en el Peru**. *Revista Peruana de Medicina de Experimental y Salud Publica* 2013, **30**(2):303-307.

4. Li X, Zhu J, Wang Y, Mu D, Dai L, Zhou G, Li Q, Wang H, Li M, Liang J: **Geographic and urban-rural disparities in the total prevalence of neural tube defects and their subtypes during 2006-2008 in China: a study using the hospital-based birth defects surveillance system**. *BMC public health* 2013, **13**:161.

5. Mansour RT, Abou-Setta AM: **Assisted reproductive technology in Egypt, 2001: Results generated from the Egyptian IVF registry**. *Middle East Fertility Society Journal* 2005, **10**(2):87-93.

6. Gonzales GF, Tapia V, Gasco M, Carrillo CE: **Maternal hemoglobin concentration and adverse pregnancy outcomes at low and moderate altitudes in Peru**. *Journal of Maternal-Fetal and Neonatal Medicine* 2012, **25**(7):1105-1110.

7. Welaga P, Moyer CA, Aborigo R, Adongo P, Williams J, Hodgson A, Oduro A, Engmann C: **Why are babies dying in the first month after birth? A 7-year study of neonatal mortality in northern Ghana**. *PLoS ONE* 2013, **8**(3):e58924.

8. Odhiambo FO, Laserson KF, Sewe M, Hamel MJ, Feikin DR, Adazu K, Ogwang S, Obor D, Amek N, Bayoh N *et al*: **Profile: The KEMRI/CDC health and demographic surveillance system-Western Kenya**. *International Journal of Epidemiology* 2012, **41**(4):977-987.

9. Ba-Saddik IA: **Childhood cancer in Aden, Yemen**. *Cancer Epidemiology* 2013, **37**(6):803-806.

10. Scott JAG, Bauni E, Moisi JC, Ojal J, Gatakaa H, Nyundo C, Molyneux CS, Kombe F, Tsofa B, Marsh K *et al*: **Profile: The Kilifi health and demographic surveillance system (KHDSS)**. *International Journal of Epidemiology* 2012, **41**(3):650-657.

11. Larsson EC, Thorson AE, Pariyo G, Waiswa P, Kadobera D, Marrone G, Ekstrom AM: **Missed Opportunities: barriers to HIV testing during pregnancy from a population based cohort study in rural Uganda**. *PLoS ONE* 2012, **7**(8):e37590.

12. Bjerregaard-Andersen M, Biering-Sorensen S, Gomes GM, Bidonga A, Jensen DM, Rodrigues A, Christensen K, Aaby P, Beck-Nielsen H, Benn CS *et al*: **Infant twin mortality and hospitalisations after the perinatal period - a prospective cohort study from Guinea-Bissau**. *Tropical Medicine & International Health* 2014, **19**(12):1477-1487.

13. Bjerregaard-Andersen M, Gomes MA, Joaquim LC, Rodrigues A, Jensen DM, Christensen K, Benn CS, Aaby P, Beck-Nielsen H, Sodemann M: **Establishing a twin registry in Guinea-Bissau**. *Twin Research and Human Genetics* 2013, **16**(1):179-184.

14. Li Q, Hu Y, Zhong YP, Chen YP, Tang XW, Guo J, Shen LZ: **Using the Immunization Information System to Determine Vaccination Coverage Rates among Children Aged 1-7 Years: A Report from Zhejiang Province, China**. *Int J Environ Res Public Health* 2014, **11**(3):2713-2728.

15. Labrique AB, Christian P, Klemm RD, Rashid M, Shamim AA, Massie A, Schulze K, Hackman A, West KP: **A cluster-randomized, placebo-controlled, maternal vitamin A or beta-carotene supplementation trial in Bangladesh: design and methods**. In: *Trials.* vol. 12; 2011: 102.

16. Chi BH, Vwalika B, Killam WP, Wamalume C, Giganti MJ, Mbewe R, Stringer EM, Chintu NT, Putta NB, Liu KC *et al*: **Implementation of the Zambia Electronic Perinatal Record System for comprehensive prenatal and delivery care**. *International Journal of Gynecology & Obstetrics* 2011, **113**(2):131-136.

17. Blank A, Prytherch H, Kaltschmidt J, Krings A, Sukums F, Mensah N, Zakane A, Loukanova S, Gustafsson LL, Sauerborn R *et al*: **"Quality of prenatal and maternal care: bridging the know-do gap" (QUALMAT study): an electronic clinical decision support system for rural Sub-Saharan Africa**. *Bmc Medical Informatics and Decision Making* 2013, **13**.

18. Nilsen C, Ostbye T, Daltveit AK, Mmbaga BT, Sandoy IF: **Trends in and socio-demographic factors associated with caesarean section at a Tanzanian referral hospital, 2000 to 2013**. *International Journal for Equity in Health* 2014, **13**(87).

19. Chiwanga ES, Massenga G, Mlay P, Obure J, Mahande MJ: **Maternal outcome in multiple versus singleton pregnancies in Northern Tanzania: a registry-based case control study**. *Asian Pacific Journal of Reproduction* 2014, **3**(1):46-52.

20. Mahande MJ, Daltveit AK, Obure J, Mmbaga BT, Masenga G, Manongi R, Lie RT: **Recurrence of preterm birth and perinatal mortality in northern Tanzania: Registry-based cohort study**. *Tropical Medicine and International Health* 2013, **18**(8):962-967.

21. Mahande MJ, Daltveit AK, Mmbaga BT, Obure J, Masenga G, Manongi R, Lie RT: **Recurrence of perinatal death in Northern Tanzania: A registry based cohort study**. *BMC Pregnancy and Childbirth* 2013, **13**(166).

22. Mmbaga BT, Lie RT, Olomi R, Mahande MJ, Olola O, Daltveit AK: **Causes of perinatal death at a tertiary care hospital in Northern Tanzania 2000-2010: a registry based study**. *Bmc Pregnancy and Childbirth* 2012, **12**.

23. Sorbye IK, Vangen S, Oneko O, Sundby J, Bergsjo P: **Caesarean section among referred and self-referred birthing women: a cohort study from a tertiary hospital, northeastern Tanzania**. *Bmc Pregnancy and Childbirth* 2011, **11**.

24. Mmbaga BT, Lie RT, Olomi R, Mahande MJ, Kvale G, Daltveit AK: **Cause-specific neonatal mortality in a neonatal care unit in Northern Tanzania: a registry based cohort study**. *BMC Pediatrics* 2012, **12**(116).

25. Mmbaga BT, Lie RT, Kibiki GS, Olomi R, Kvale G, Daltveit AK: **Transfer of newborns to neonatal care unit: A registry based study in Northern Tanzania**. *BMC Pregnancy and Childbirth* 2011, **11**(68).

26. Habib NA, Wilcox AJ, Daltveit AK, Basso O, Shao J, Oneko O, Lie RT: **Birthweight, preterm birth and perinatal mortality: a comparison of black babies in Tanzania and the USA**. *Acta Obstet Gynecol Scand* 2011, **90**(10):1100-1106.

27. Bergsjo P, Mlay J, Lie RT, Lie-Nielsen E, Shao JF: **A medical birth registry at Kilimanjaro Christian Medical Centre**. *East African journal of public health* 2007, **4**(1):1-4.

28. Zegers-Hochschild F, Masoli D, Schwarze JE, Iaconelli A, Borges E, Pacheco IM: **Reproductive performance in oocyte donors and their recipients: Comparative analysis from implantation to birth and lactation**. *Fertility and Sterility* 2010, **93**(7):2210-2215.

29. Moshabela M, Sene M, Nanne I, Tankoano Y, Schaefer J, Niang O, Sachs SE: **Early detection of maternal deaths in Senegal through household-based death notification integrating verbal and social autopsy: a community-level case study**. *BMC Health Serv Res* 2015, **15**:9.

30. Ngana FR, Myers BA, Belton S: **Health reporting system in two subdistricts in Eastern Indonesia: Highlighting the role of village midwives**. *Midwifery* 2012, **28**(6):809-815.

31. Liu L, Li M, Yang L, Ju L, Tan B, Walker N, Bryce J, Campbell H, Black RE, Guo Y: **Measuring coverage in MNCH: a validation study linking population survey derived coverage to maternal, newborn, and child health care records in rural China**. *PLoS ONE* 2013, **8**(5):e60762.

32. Bari S, Mannan I, Rahman MA, Darmstadt GL, Serajil MH, Baqui AH, Arifeen S, Rahman SM, Saha SK, Ahmed AS *et al*: **Trends in use of referral hospital services for care of sick newborns in a community-based intervention in Tangail District, Bangladesh**. In: *Journal of health, population, and nutrition.* vol. 24; 2006: 519-529.

33. Goudar SS, Carlo WA, McClure EM, Pasha O, Patel A, Esamai F, Chomba E, Garces A, Althabe F, Kodkany B *et al*: **The Maternal and Newborn Health Registry Study of the Global Network for Women's and Children's Health Research**. *International Journal of Gynaecology & Obstetrics* 2012, **118**(3):190-193.

34. Gisore P, Shipala E, Otieno K, Rono B, Marete I, Tenge C, Mabeya H, Bucher S, Moore J, Liechty E *et al*: **Community based weighing of newborns and use of mobile phones by village elders in rural settings in Kenya: a decentralised approach to health care provision**. *BMC pregnancy and childbirth* 2012, **12**:15.

35. Belizan JM, McClure EM, Goudar SS, Pasha O, Esamai F, Patel A, Chomba E, Garces A, Wright LL, Koso-Thomas M *et al*: **Neonatal death in low- to middle-income countries: A global network study**. *American Journal of Perinatology* 2012, **29**(8):649-655.

36. Bang A, Bellad R, Gisore P, Hibberd P, Patel A, Goudar S, Esamai F, Goco N, Meleth S, Derman RJ *et al*: **Implementation and evaluation of the Helping Babies Breathe curriculum in three resource limited settings: does Helping Babies Breathe save lives? A study protocol**. *BMC Pregnancy & Childbirth* 2014, **14**:116.

37. Thompson A, Castle E, Lubeck P, Makarfi PS: **Experience implementing OpenMRS to support maternal and reproductive health in Northern Nigeria**. *Studies in Health Technology and Informatics* 2010, **160 (PART 1)**:332-336.

38. Siika AM, Rotich JK, Simiyu CJ, Kigotho EM, Smith FE, Sidle JE, Wools-Kaloustian K, Kimaiyo SN, Nyandiko WM, Hannan TJ *et al*: **An electronic medical record system for ambulatory care of HIV-infected patients in Kenya**. *International Journal of Medical Informatics* 2005, **74**(5):345-355.

39. Dongmo R, Fenieys D, Aminou M, Calvez T, Gruenais ME, Thonneau P: **Introduction of an obstetric health information system: Results of a pilot study in North Cameroon**. *Revue d'Epidemiologie et de Sante Publique* 2006, **54**(6):507-515.

40. Chhetri S, Shrestha NR, Pilgrim T: **Pregnancy complicated by heart disease in Nepal**. *Heart Asia* 2014, **6**(1):26-29.

41. Mehta U, Clerk C, Allen E, Yore M, Sevene E, Singlovic J, Petzold M, Mangiaterra V, Elefant E, Sullivan FM *et al*: **Protocol for a drugs exposure pregnancy registry for implementation in resource-limited settings**. *BMC pregnancy and childbirth* 2012, **12**:89.

42. Luhm KR, Cardoso MRA, Waldman EA: **Vaccination coverage among children under two years of age based on electronic immunization registry in Southern Brazil.** *Revista de Saude Publica* 2011, **45**(1):90-98.

43. Pandian JD, Venkateswaralu K, Thomas SV, Sarma PS: **Maternal and fetal outcome in women with epilepsy associated with neurocysticercosis**. *Epileptic Disorders* 2007, **9**(3):285-291.
